# Contribution of Various Loads to the Convex Shape of Rock Wool Insulation Slabs during Production

**DOI:** 10.3390/ma16196596

**Published:** 2023-10-08

**Authors:** Jurij Hladnik, Boris Jerman

**Affiliations:** Faculty of Mechanical Engineering, University of Ljubljana, Aškerčeva 6, 1000 Ljubljana, Slovenia; jurij.hladnik@fs.uni-lj.si

**Keywords:** chain conveyor, finite element analysis, stone wool, response surface optimization, design of experiments, elevated temperatures, inverse problem, thermo-mechanical loading, non-linear temperature

## Abstract

Rock wool insulation slabs are produced in special curing ovens, where molten rock wool fibres coated with binder are compressed between two slat conveyors and blown with hot air for vitrification. Often, the cross-section of the final slabs is slightly convex, which is undesirable. The degree of convexity depends on the deformation of the steel crossbars of the slat conveyors, which are subjected to combined pressure and nonlinear temperature loadings. Due to this complex loading state, it is difficult to determine the contribution of individual load to the total deformation. The main aim of the study was to determine these contributions. Temperature and stress measurements of the crossbars were performed during rock wool production. Upon collecting these measurements, a finite element (FE) model of a crossbar was established for the identification of the pressure loading acting on the crossbars, and finally for determination of their deformations. As a main result of the study, an inverse problem-based methodology for the identification of the deflection of a structure due to unknown temperature and pressure loadings was established and applied on the specific case. The deviations between the deformations of the FE crossbars and the final shape of the rock wool slabs were below 10%, which validates the novel methodology.

## 1. Introduction

**Figure 1:**
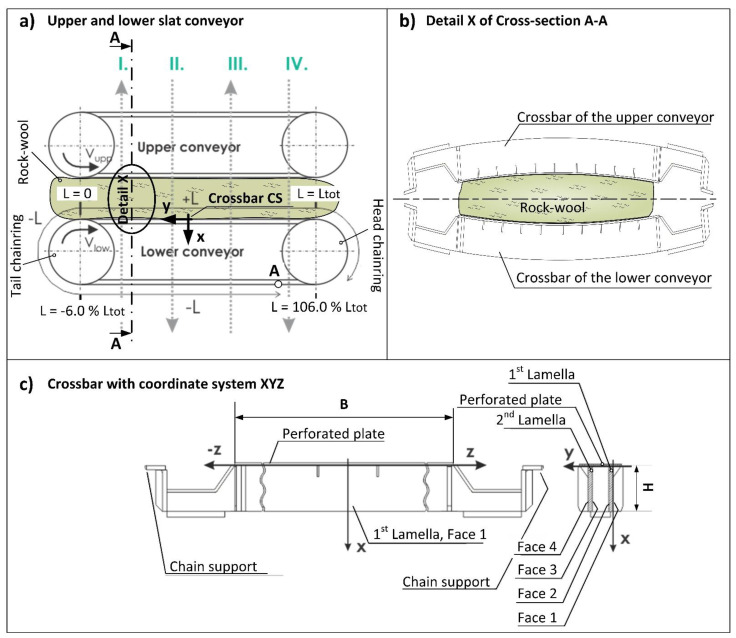
(**a**) Upper and lower slat conveyor inside the curing oven with designated *L* coordinate along the lower conveyor and local coordinate system XYZ (Crossbar CS) of an arbitrary crossbar. The grey dotted arrows represent the hot air blowing directions (I., II., III. and IV.), point A represents the position where the measuring instrument was stopped, and the sensors were reconnected. (**b**) Exaggerated deformation of the crossbars leading to the convex shape of the rock wool. (**c**) A crossbar with annotated main parts and its corresponding local coordinate system XYZ.

Rock wool (also stone wool) is a fibrous material made from molten rock and is used especially as thermal insulation for buildings [1]. It is made from various rock types such as limestone, dolomite and diabase which are melted into a magma-like liquid that is exposed to a thin jet of air or steam that breaks the liquid into long strands of fibres [2]. During this process, the binder with water is added so that the fibres vitrify [1]. The fibres are then spread out and compressed between two slat conveyors (Figure 1a,b) which pass through a curing oven. Here, hot air is blown through the rock wool fibres to allow the binder to cure [3]. To achieve adequate thickness and condensation of the rock wool through its thickness, an appropriate distance between the slat conveyors is set. To achieve adequate condensation of the rock wool in its longitudinal direction, slightly different velocities of the conveyors are chosen. Since the geometry of the conveyor crossbars, which compress and push the rock wool through the curing oven, affects the final geometry of the rock wool slabs, it is important that they stay as straight as possible during this process.

A common problem of the described rock wool production lines is that the thickness of the rock wool slabs exiting the curing oven is slightly convex along their width. The convex-shaped cross-section of the slabs appears due to the bending deformation of the slat-conveyor crossbars (Figure 1b). These steel crossbars bend partly due to the mechanical loading (rock wool pressure and their own weight) and partly due to the temperature loading of the blown hot air inside the curing oven. In order to minimize the convexity of the slabs, it is necessary to know the contribution of individual causes to the overall deformation of the crossbars, since design improvements may differ depending on the deformation cause. It must be pointed out that other causes may also affect the final rock wool geometry, but not its convexity, and can therefore be ignored in the present study. Since the own weight of the crossbars has a negligible influence on its deformation, and even lesser on the convexity of the slabs, it was also not considered in the study.

Multiple studies on combined temperature and mechanical loading influence on the stress–strain state of steel [4] and other constructions [5] were performed in the past, especially concerning fire safety. Although mechanically and thermally loaded systems have been analytically solved for various complex situations, e.g., [6,7], real-life examples usually become too complex for analytical solving and therefore experimental, in combination with numerical methods, are often used [8]. e.g., Lausova et al. [9,10] performed an experiment validated by numerical finite element (FE) simulation of a steel frame structure under fire loading, which caused a non-uniform temperature load over the cross-section and thus additional internal forces. Lausova et al. [11] also conducted an experimental–numerical study of the non-uniform temperature distribution across hollow steel sections of different sizes under fire loading. Moreover, Mourao and da Silva [12] analysed the behaviour of steel beams under uniform temperature rising. In this study, typical examples of beams loaded with continuous loads were analysed using the FE method.

Mechanical and non-uniform temperature loadings (due to solar radiation) are also present in bridge constructions [13,14] and in insulation composite sandwich wall panels for buildings [15]. Moreover, bowing of concrete sandwich panels, used for building and thermal insulation, due to the temperature difference on the inner and outer side, has also been investigated [16].

However, not many studies have been found on slat or chain conveyors specifically [17], nor on the stress–strain analyses of their crossbars, while no literature dealing with the bending deformation of the slat conveyor crossbars due to the thermal–mechanical loading was found. The most related study, dealing with the stress–strain analysis of slat conveyors, was the study of Tsonev [18], where an FE analysis of a chain link of a slat conveyor for rock material transportation was performed to identify the reasons for its destruction. Here, the FE model of the chain link was verified by experimental stress measurements.

The main goals of the present study were to determine the contribution of unknown pressure and non-linear thermal loading to the total bending deformation of a slat-conveyor crossbar in a specific rock wool production line, and to establish a methodology for its determination. Other goals were to compare the bending deformation of the crossbars with the final convex deformation of the rock wool slabs, and thus to verify the method validity, and to present suggestions for minimization of the crossbar deformations.

## 2. Methods

### 2.1. Measurements

**Figure 2:**
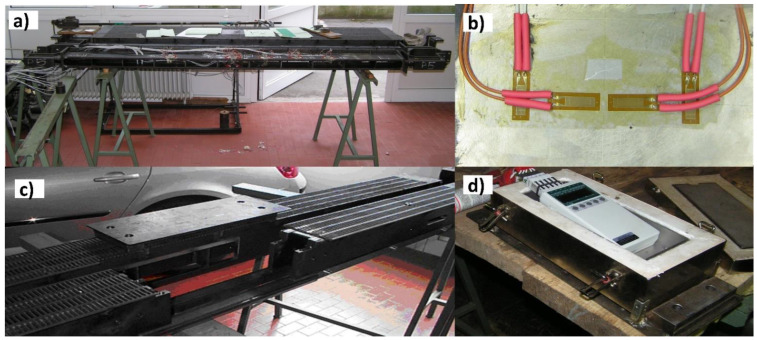
Preparation of the crossbars in the laboratory. (**a**) The stress crossbar equipped with strain gauges. (**b**) Close view of a full-bridge strain gauge sensor on the stress crossbar. (**c**) Processed crossbar for insertion of the data acquisition measuring instrument. (**d**) The data acquisition measuring instrument embedded in the insulation box.

Stress and temperature measurements were performed on the steel (S235, EN 10025 [19]) crossbars of the lower slat conveyor during the production of rock wool slabs (approximately, density *ρ* = 130 kg/m^3^ and thickness *d* = 45 mm). One of the crossbars (stress crossbar, Figure 2a) was equipped with 14 full-bridge strain gauge sensors (Figure 2b) for high temperatures (type ZFLA-6-350-1, Tokyo Measuring Instruments Laboratory Co., Ltd., Tokyo, Japan) denoted with MP01 up to MP14. These sensors actually measured the changes in the output voltage in the Wheatstone bridges, which appeared due to material deformation, independently of the temperature changes.

A neighbouring crossbar (temperature crossbar) was equipped with 17 platinum sheathed thermocouples (ELPRO 2220 6620, ELPRO Lepenik & Co., Ltd., Miklavž na Dravskem Polju, Slovenia) placed inside the drilled holes in the crossbars. These sensors were denoted with MP15 up to MP31. Because of the exposure to elevated temperatures (*T_max_* ≈ 200 °C), all the measuring equipment had to be adequately protected or designed for such conditions.

For the description of the sensor positions on the crossbars, a local coordinate system XYZ was defined (Figure 1c). The strain sensor locations on the crossbars are given in Table 1, while the temperature sensor locations are given in Table 2. These locations were carefully selected to provide a complete picture of the temperature and stress state of the crossbars. For the description of the crossbar position along the lower slat conveyor, a coordinate *L* was introduced with its origin at the top of the tail chainring (Figure 1a). The coordinate *L* increased along the upper branch of the lower conveyor and decreased (negative value) along its lower branch.

To enable the measurement, three neighbouring crossbars also had to be processed. In one of them, a portable multi-function data acquisition measuring instrument (MA 2390-8, Almemo; Ahlborn Mess- und Regelungstechnik GmbH, Holzkirchen, Germany, Figure 2c) was embedded in a temperature-insulated box (ZB1030IB, Almemo, Figure 2d). The construction changes made on the three neighbouring and the two measuring crossbars neither influenced the loading state, nor the deformation of the measuring crossbars.

Since a measuring instrument with more measuring channels did not fit into the insulation box, whose size was limited by the available space, the stress and temperature measurements were performed separately, one after another, in groups of five sensors at once. For the validity of the measurements, stable operating conditions in the curing oven were of essential importance. These were achieved by inserting the processed crossbars without the measuring instruments into the slat conveyor during the overhaul of the curing oven and by starting the measurements after a few days of continuous production of the insulation slabs. For the same reason, the measurement procedure was organized in such a way that the stops of the production process during the measurements lasted less than 5 min. The stops of the measuring instrument were always performed at the same place (point A in Figure 1a).

In order to prevent higher temperatures inside the insulated box than the allowable operating temperatures of the measuring instrument, the measuring time was limited to two full turns (approximately 20 min) of the lower conveyor. Between the successive measurements, the conveyors were stopped, the insulated box was removed and cooled with compressed air, and the measured data were saved to a PC. In the meantime, the conveyors were restarted so the rock wool production was not interrupted for too long and the operating conditions did not change considerably. Before the next set of measurements was taken, the conveyors were stopped once again, the measuring instrument was connected with the next set of sensors and returned to its measuring position together with the cooled insulation box.

The curing oven is divided into more heating zones, where the blowing directions of hot air are altered (Figure 1a). The blowing directions alter with the purpose to evenly heat the rock wool from both sides, and thus to achieve more uniform properties of the insulation slabs.

#### 2.1.1. Stress Sensor Calibration

For calibration of the stress sensors, the stress crossbar was removed from the slat conveyor, simply supported at its chain supports, and loaded with a known bending moment by adding weights at its centre in 14 steps up to a total load of 255.4 kg. Meanwhile, the output voltage of the stress sensors was sampled and compared with the theoretical longitudinal (bending) stress at the individual *i*-th sensor:

(1) $$\sigma_{zz,i}=\frac{m_{j}\cdot g\cdot z_{i}}{2\cdot I_{y}}\cdot e_{i}$$

derived after the basic bending stress equation. In this equation $m_{j}$ is the *j*-th cumulative mass of the applied weights in kg, *g* the gravitational acceleration of 9.81 $\frac{m}{s^{2}}$, $z_{i}$ the distance between the *i*-th sensor centre and the closer support of the crossbar in the local *z*-direction in mm, $e_{i}$ the distance between the *i*-th sensor centre to the neutral bending axis of the crossbar in the local *x*-direction in mm, and $I_{y}$ the second moment of area of the crossbar cross-section around the local *y*-axis in ${mm}^{4}$.

Since the relationships between the output voltages and the calculated longitudinal stresses $\sigma_{zz}$ at the sensors were linear, the conversion factors of the output voltages into stresses were defined as the direction coefficients of the linear stress–voltage approximation curves for each sensor individually.

#### 2.1.2. Temperature Sensor Accuracy

All the temperature sensors were placed at once into a high-precision electric oven (XU024, France Etuves, Chelles, France) for their accuracy check at three different temperatures of 100 °C, 250 °C and 275 °C. Their corresponding relative standard deviations at these temperatures were ±0.054%, 0.036% and 0.043%, respectively.

#### 2.1.3. Data Processing

The data from the second measurement turn of the lower conveyor were used for the analysis, when the temperatures in the curing oven were stabilized. The time axis of the results was replaced with the location of the observed crossbar along the lower conveyor expressed as a portion of *L_tot_*, which was determined from the velocity of the conveyor.

The measurements began with the measurement crossbars turned down (point A in Figure 1a). The influence of the gravity action direction change had to be considered in the measured stress results, when the stress crossbar was turned up. This was carried out by subtraction of the output voltage change, measured at the turnaround of an empty crossbar, from the sensor output voltages acquired at the upper part of the conveyor.

It was presumed, that the deformations of the crossbars in the last heating zone of the curing oven reflected the final geometry of the rock wool slabs, including the undesired convexity. Therefore, the middle third of this zone (denoted as the deeper observed area; DOA) between 77% and 89% of *L_tot_* was analysed in greater detail.

### 2.2. Finite Element Model

To identify the actual pressure loading acting on the crossbars due to the rock wool pressure, and to quantify their temperature and pressure loading deformations in the DOA, an FE model of a crossbar was established in commercially available software Ansys 18.2 Workbench (Ansys, Inc., Canonsburg, PA, USA). The perforated plate, welded on the top of the lamellas (Figure 1c), was simplified as a full rectangular plate. Since this plate is compressively loaded in its longitudinal direction due to bending of the crossbar, the modelled full plate had to have the same compressive characteristics as the real-life perforated plate. These were achieved through the usage of a reduced thickness, which was calculated upon the results of an additional FE analysis of a standalone perforated plate loaded in compression:

(2) t=(F·B)/(b·E·∆B) 

derived after basic linear stress equations. In this equation ∆B is the longitudinal contraction of the perforated plate, *B* its length, *b* width, *E* its Young’s modulus and *F* the compressive load.

The material was set to elastic isotropic steel with a Young’s modulus of $2.1\times 10^{5}\;MPa$, Poisson’s ratio of 0.3 and temperature expansion coefficient of $1.2\times 10^{-5\;}K^{-1}$. Since the Young’s modulus of constructional steel reduces for a maximum of 10% at 200 °C [20], as much as the temperatures of the crossbars maximally rise in the curing oven, its temperature dependency was not encountered in the model.

The 3D geometry model of the crossbar was meshed with 52,463 FEs, mainly 20-node hexahedrons. The mesh quality characterised by the Ansys built-in tool was on average 0.90. The welded connections between the adjacent parts of the crossbar were represented by automatically established bonded surface-to-surface contacts.

#### 2.2.1. Boundary Conditions and Pressure-Loading Determination

**Figure 3:**
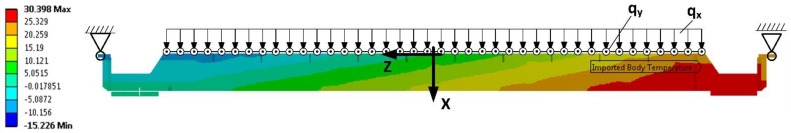
FE model of a crossbar loaded with uniform pressure $q_{x}$ and $q_{y}$, and with a non-uniform temperature filed. The temperature loading presented in °C was established upon the average temperature differences measured in the deeper observed area.

The FE model of the crossbar (Figure 3) was simply supported at its chain supports and loaded with a uniform initial pressure loading ${\overset{⃑}{q}}_{0}=\left(q_{x0},\;q_{y0}\right)$ on the upper surface of the simplified perforated plate. Besides that, it was loaded with a temperature field obtained from the average measured temperature differences in the DOA of the slat conveyor (see Section 3.2). The temperature loading was entered into the FE model by 1000 equally distributed points over the midplane of each lamella. At these points, the temperature loading was linearly distributed over the whole FE model.

The initial pressure loading ${\overset{⃑}{q}}_{0}$ had to be reasonably estimated and consisted of two components. The first $q_{x0}$, represented the pressure loading of the rock wool normal to the surface of the perforated plate, while the second $q_{y0}$, represented the shear loading of the rock wool due to the velocity differences between the two slat conveyors. The actual pressure loading $\overset{⃑}{q}=\left(q_{x},\;q_{y}\right)$ was found by solving an inverse problem by the Response surface optimization, a module integrated in Ansys. The optimization objective was set to minimum standard deviation (STD) between the average measured and simulated longitudinal stresses in MP02, MP07, MP09 and MP14, which were chosen for the representation of the stress state of the crossbar.

In order to use the Response surface optimization, the relations between the input and output optimization parameters had to be well approximated with response surfaces. This was carried out by solving the FE model for a set of $q_{x}$ and $q_{y}$ values determined by the Central Composite design method. After calculating the reference points, 2nd order polynomial response surfaces were created and used in the Nonlinear Programming by Quadratic Lagrangian optimization method.

## 3. Results and Discussion

### 3.1. Crossbar Temperatures along the Curing Oven

**Figure 4:**
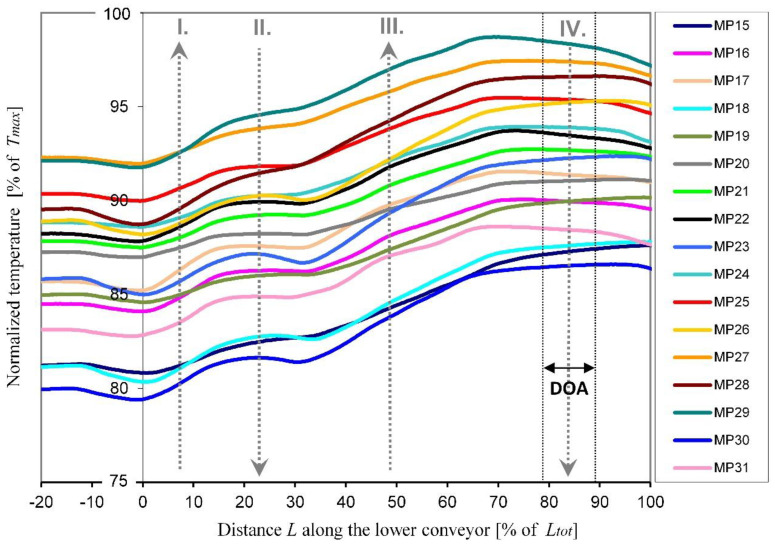
Normalized temperatures in the temperature crossbar along part of the lower conveyor (2nd measurement turn). MP—measuring point; DOA—deeply observed area. The dotted grey arrows designate the hot air blowing directions (I., II., III. and IV.). The distance along the conveyor is expressed as a portion of the total conveyor’s length *L_tot_* (see Figure 1a).

In Figure 4, the normalized temperatures in the temperature crossbar are presented for a part of the second measurement turn along the lower conveyor. At the lower branch, up to −10% of *L_tot_* the temperatures in all sensors were stable. Afterwards, a small temperature decrease at the tail chainring was present with a minimum at *L* = 0, due to the boundaries between the curing oven and the environment. Hereon, the temperatures increased in all sensors up to their maximum at 70–90% of *L_tot_*. At the end of the curing oven, the temperatures slightly decreased due to its boundaries.

From the temperature courses, the influence of the hot air blowing direction is recognized. When the air was blown up (from the lower to the upper conveyor), the temperature gradients were higher than in the case when the air was blown down (from the upper to the lower conveyor) when the gradients were lower, or even negative. This is because in the first case, the hot air directly heated the lamellas and the temperature sensors while in the second case, the hot air had to pass through the upper conveyor and the layer of rock wool before it reached the crossbars and the sensors. In the DOA, the hot air was blown upside down, which is one of the reasons why the measured temperatures did not change much in this area; some remained constant, some slightly increased and some even slightly decreased.

### 3.2. Crossbar Temperature Field

**Figure 5:**
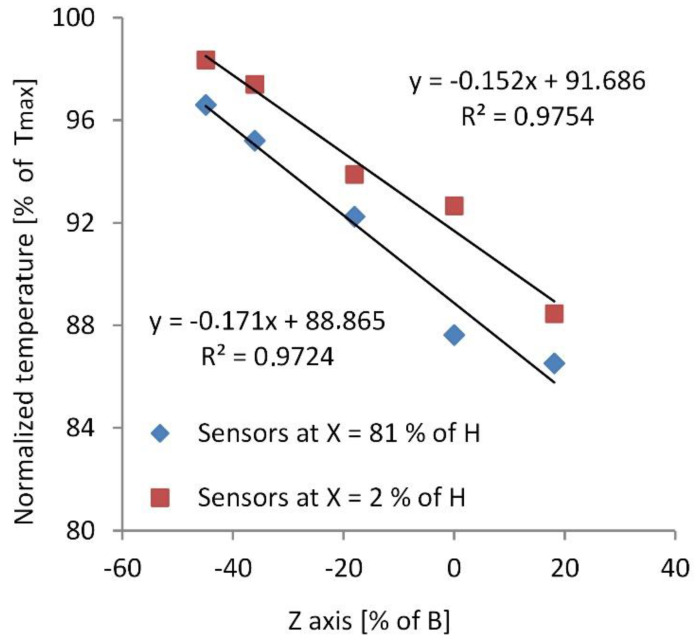
Dependence of the average normalized temperatures along the *z*-direction of the crossbar (2nd lamella) at two different *x* locations in the deeper observed area. The *x* and *z* dimension are presented as portion of *H* and *B*, respectively (see Figure 1c).

To establish the temperature field for the FE model, the temperature course had to be analysed in all main directions of the crossbar in the DOA. In Figure 5, the average normalized temperatures in dependence from the z-direction of the crossbar $T\left(z\right)$ are presented for the second lamella at two different x locations; x = 2% of *H* (sensors MP18, MP23, MP26, MP28 and MP30) and x = 81% of *H* (sensors MP21, MP24, MP25, MP27, MP29 and MP31). These temperatures decreased in the positive z-direction with a similar gradient for both x locations. The average temperature difference between the opposite ends of the perforated plate (at $z=-\frac{B}{2}$ and at z = $+\frac{B}{2}$), obtained from $T\left(z\right)$ extrapolation, accounted for $∆T_{2\%H}\left(z\right)=$30.4 °C at x = 2% of *H*, and $∆T_{81\%\;H}\left(z\right)=$34.2 °C at *x* = 81% of *H*. Since the crossbars may be treated as simply supported beams which allow some longitudinal deformation in the *z*-direction, these $∆T\left(z\right)$ temperature differences did not contribute to the bending of the crossbars.

**Figure 6:**
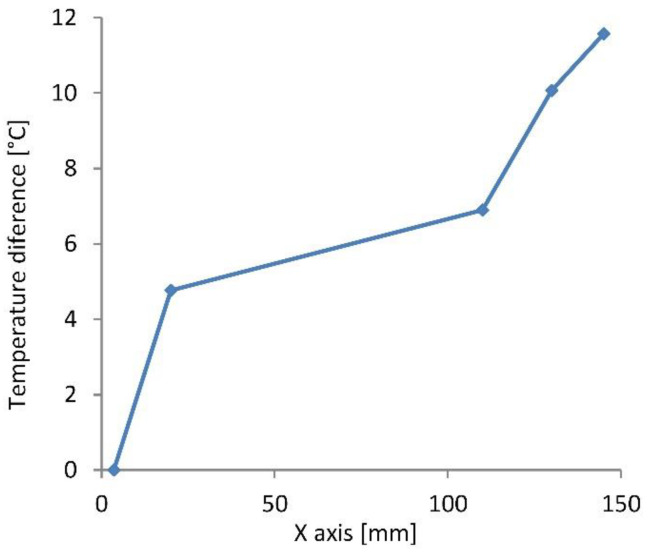
Average temperature difference in the middle of the 2nd lamella (z = 0) depending on the *x*-direction of the crossbar in the deeper observed area. The *x* dimension is presented as a portion of *H* (see Figure 1c).

In Figure 6 the average temperature difference along the *x*-direction of the crossbar $∆T\left(x\right)$ is presented for the 2nd lamella at *z* = 0 (sensors MP18 to MP22). The lowest temperatures appeared in the sensor closest to the perforated plate and rose in the positive *x*-direction. In consideration of the $∆T\left(z\right)$ dependency, the temperature difference $∆T\left(x\right)$ was scaled along the *z*-direction. As a result, a temperature field $∆T\left(x,z\right)$ was obtained for the second lamella. The temperature differences in this field in the *x*-direction (between *x* = 2–81% of *H*) accounted for 9.6 °C at z = +*B*/2 and 13.5 °C at z = −*B*/2. In contrast to the $∆T\left(z\right)$, the $∆T\left(x\right)$ temperature differences contributed to the bending deformation of the crossbars around the *y*-axis, causing displacements in the *x*-direction.

In the second lamella, 14 temperature sensors were mounted to measure the temperature distribution in the *x* and *z* direction, while in the first lamella, only three temperature sensors were mounted to measure the temperature difference along the *y*-direction. From the comparison of the average temperatures of the same *x*-*z* lying sensors in the first and second lamella (MP15 vs. MP19, MP17 vs. MP24 and MP16 vs. MP21), an average temperature difference $∆T\left(y\right)$ of 5.3 ± 0.2 °C was obtained in the DOA. The relatively small standard deviation of $∆T\left(y\right)$ confirms a similar $∆T\left(x,z\right)$ temperature field also in the first lamella. Therefore, the first lamella in the FE model was loaded with the same temperature field as the second lamella, reduced by 5.3 °C.

### 3.3. Stress Measurement Results

**Figure 7:**
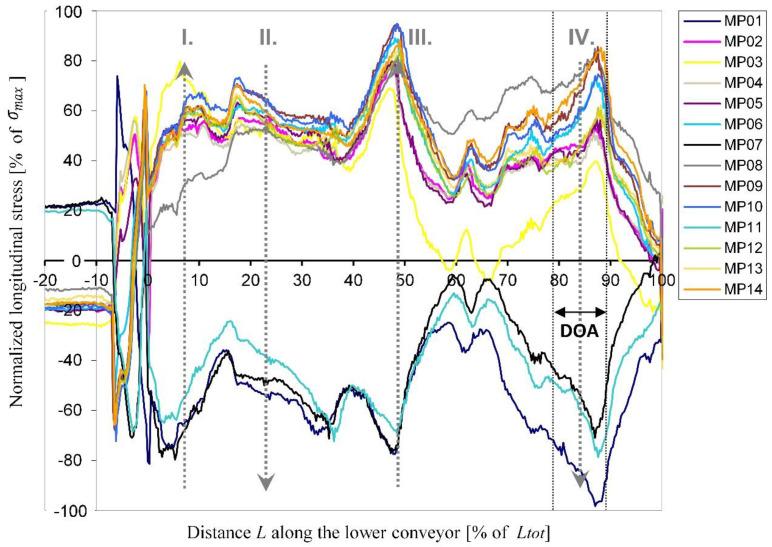
Normalized longitudinal stresses $\sigma_{zz}$ in the stress crossbar along part of the lower conveyor (2nd measurement turn). MP—measuring point, DOA—deeply observed area. The dotted grey arrows designate the hot air blowing directions (I., II., III. and IV.). The distance along the conveyor is expressed as a portion of the total conveyor’s length *L_tot_* (see Figure 1a).

In Figure 7, the normalized longitudinal stresses $\sigma_{zz}$ in the stress crossbar are presented along the same part of the lower conveyor as the temperature results (Figure 4). These stresses are already corrected for the influence of the own weight of the crossbars (see Section 2.1.3). At the lower branch, up to approximately −7.0% of *L_tot_*, the stresses in all sensors were stable. At the tail chainring, at the transition from the lower to the upper conveyor branch (−7.0% *L_tot_* ≤ *L* ≤ 0), the stresses in all sensors oscillated and changed their sign due to the turnaround of the crossbar. Here, stress peaks were observed due to dynamic effects.

At the beginning of the upper branch of the conveyor (*L* = 0), the absolute stress values of all sensors increased due to the pressure of the dosed rock wool. Along the upper conveyor branch (*L* > 0) these stresses reached four significant peaks which coincided with the centres of the heating zones and most probably appeared due to smaller distances between the conveyors at these areas.

For positive *L* values, the stresses in sensors MP01, MP07 and MP11 were compressive while in other sensors, tensile stresses appeared due to their opposite position to the neutral bending axis of the crossbar. All the tensile and compression stresses followed a similar course.

From the stress comparison between the sensors MP02 vs. MP09, MP01 vs. MP07 and MP05 vs. MP06, which lie on identical *x*-*z* positions but on different lamellas, the influence of the horizontal pressure $q_{y}$ and of the temperature difference $∆T\left(y\right)$ was recognized. The tensile stresses in the sensors on the second lamella were higher than the stresses of their duplicates on the first lamella, which indicates the bending of the crossbars around their *x*-axis, causing displacements in the *y*-direction (for the direction definition, see Figure 1). These results agree with the fact that the lower conveyor moves at a slightly higher velocity than the upper conveyor. The maximum stresses were ~11 MPa, which confirms the adequacy of the elastic material model.

### 3.4. Finite Element Model Results

The actual pressure loading $\overset{⃑}{q}$ acting on the perforated plate of the crossbar was determined upon the FE model of the crossbar and the Response surface optimization (see Section 2.2.1). The quality of the response surfaces created upon multiple FE model simulations for various input parameters was estimated as “good” by the default Ansys evaluation parameters; therefore, optimization upon these surfaces was carried out. The final optimal values of $q_{x}$ and $q_{y}$ were $1.25\times 10^{-2}$ MPa and $1.31\times 10^{-3}$ MPa, respectively.

**Figure 8:**
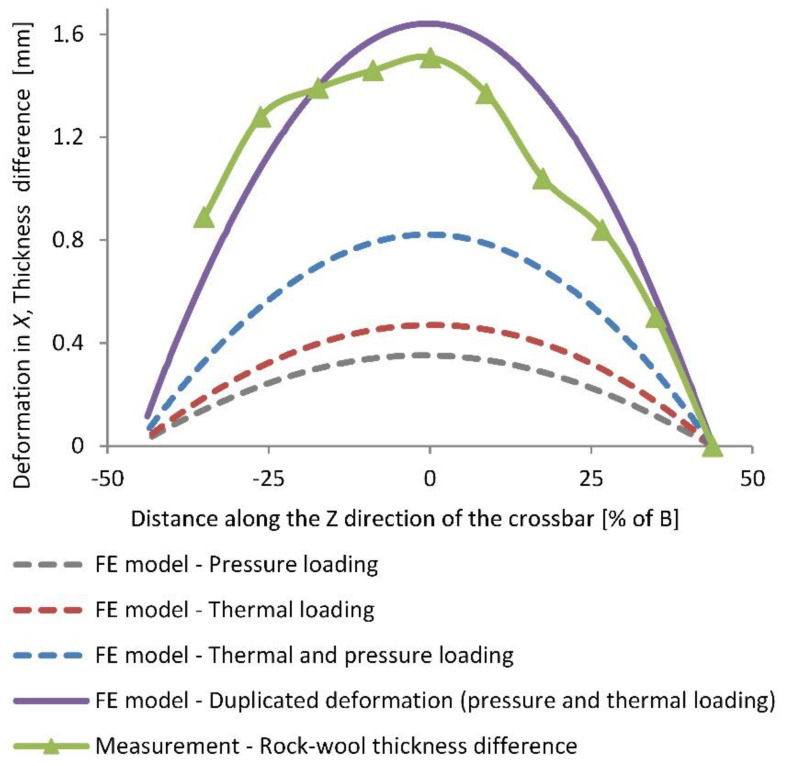
The average thickness difference of the final rock wool slabs along their transverse direction and the vertical deformation of the crossbar FE model due to temperature and pressure loading separately, combined, and duplicated.

In Figure 8, the deformation of the FE model of the crossbar in its vertical *x*-direction is presented due to temperature and pressure loading separately, and is combined along the relevant length of the perforated plate. The thermal loading contributed 57% to the maximal vertical deformation, while the pressure loading contributed the remaining 43%.

Because the deviations in thickness of the final rock wool slabs result from the deformations of the upper and lower conveyor crossbars, the vertical deformation of the crossbar FE model (in the *x*-direction of the crossbar) needed to be doubled for proper comparison with the measured thickness difference of the final rock wool slabs. By doing so, the assumption of equal deformation of lower and upper slat-conveyor crossbars was adopted. From this comparison, a good agreement between the deformation of the FE model and the actual rock wool thickness was recognized with maximum deviations of less than 10%.

From Figure 8, it is also visible that all the FE model deformations, regarding the rock wool thickness differences, were slightly asymmetrical; the maximal deformation is shifted to the negative *z* side. This happened most probably due the slightly unsymmetrical crossbar geometry (different distance between the left/right support to the beginning of the perforated plate) and due to the non-uniform temperature distribution.

### 3.5. Suggestions for Rock Wool Slab Convexity Minimization

As the convexity of the final rock wool slabs depends mainly on the bending deformation of the lower and upper slat-conveyor crossbars during the production, the crossbar deformation needs to be minimized in order to reduce the convexity of the slabs. A common solution for minimization of the crossbar deformation due to the combined pressure and temperature loading is to increase their bending stiffness. This may be achieved by increasing the section modulus of the crossbars around their *y*-axis or by changing the support configuration, e.g., by adding additional supports to the crossbars or by shifting the existing two supports towards the centre of the crossbars. Another solution adequate for both deformation causes is pre-curving of the crossbars; i.e., crossbars should have an initial curvature, which would flatten under perceived loadings. The first solution can just reduce the size of the slab convexity or divide the convex shape into smaller bumps (if the position of the supports is changed), while the latter solution could eventually eliminate the convexity.

For minimization of the crossbar deformation other solutions are also theoretically possible such as the usage of materials with greater Young’s modulus or smaller thermal expansion coefficient, or by providing a more uniform temperature environment in the curing oven.

### 3.6. Summary of the Introduced Method

A method for the determination of the contribution of unknown pressure and non-linear temperature loading to the total deformation of a slat-conveyor crossbar was introduced. The procedure of the method is summarised in the following steps.

- Temperature and stress were measured at multiple positions of the crossbar during the rock wool production in the curing oven.
- The non-linear temperature field of a crossbar was determined by interpolation/extrapolation of the average measured temperature differences between the measured positions for the relevant part of the curing oven (DOA).
- An FE model of the crossbar was established and loaded with the derived temperature field and with an initial constant pressure loading ${\overset{⃑}{q}}_{0}$ acting on the crossbar.
- The actual pressure loading ${\overset{⃑}{q}}_{}$ was determined by solving the inverse problem of the FE model by the usage of the Response surface optimization. Here, the objective was to minimize the standard deviation between the measured and simulated longitudinal stresses at specific positions on the crossbar.
- The FE model was loaded separately and combined with the obtained thermal field and pressure loading. Thus, bending deformation contributions due to each loading were obtained.

### 3.7. Limitations

One potential limitation of this study is that it was conducted solely on a specific slat conveyor, rather than on multiple structures. However, this limitation is mitigated by the fact that the crossbars of the examined slat conveyor exhibit a simple uniformly supported beam geometry. This makes them good representatives of similar simple beams commonly found in other engineering structures. Consequently, this reduces the necessity for validating the methodology across additional cases.

Moreover, there are many sources of error that can lead to the differences between the final deformation of the rock wool slabs and the deformation of the FE model of the crossbar (Figure 8): uncertainty of stress, temperature and deformation measurement, elastic material model used in the FE model, errors due to FE method, etc. However, it is believed that the largest sources of error for these differences are the approximated temperature field on the crossbars and the estimated pressure acting on the crossbars. Taking into account all these possible sources of error, the differences between the final deformation of the rock wool slabs and the deformation of the FE model of the crossbar below 10% prove the accuracy of the study and the applicability of the method used.

## 4. Conclusions

In this research, the reasons for the convex-shaped rock wool slabs were investigated. It was shown that the final geometry of the rock wool slabs depends on the shape of the upper and lower slat-conveyor crossbars between which the rock wool is compressed during production. These crossbars mainly deform due to the rock wool pressure and the temperature loading in the curing oven. The main goal of the study was thus to determine the individual contribution of the thermal and pressure loading to the total deformation of the crossbars. This was conducted using a method based on extensive temperature and stress measurements of the crossbars during the rock wool production in combination with FE analyses of a crossbar. The main findings and contributions of the study are as follows:

- A methodology for identification of the deflection of a structure due to unknown temperature and pressure loading contribution was established. This method may be used also for other similar constructions, and not only for slat conveyors in the rock wool production.
- Construction suggestions for the slat conveyors under combined pressure–thermal loadings, which lead to smaller crossbar deflections, were given. These suggestions may also be used in other similar constructions.

Other findings, that refer to the specific slat conveyor, are as follows:

- The thermal loading contributed 57% and pressure loading 43% to the total vertical deformation of the crossbars.
- The temperature distribution in the crossbars turned out to be significantly non-uniform: the average temperature difference between the opposite ends of the perforated plate in the longitudinal z-direction accounted for approximately 30.4 °C, while the average temperature difference in the vertical *x*-direction for approximately 9.6 °C at one end of the perforated plate and to 13.5 °C at the other end.
- The pressure components $q_{x}$ and $q_{y}$ acting on the perforated plate of the crossbar were evaluated as $1.25\times 10^{-2}$ MPa and $1.31\times 10^{-3}$ MPa, respectively.
- The deformation of the FE crossbar agreed well with the measured thickness difference of the rock wool slabs with differences lower than 10%, which confirms the validity of the presented method.
- For the reduction of the convex-shaped rock wool slabs, pre-curving of the crossbars and shifting of their supports towards the crossbar centre are suggested. A more uniform temperature environment in the curing oven is also suggested.

## Figures and Tables

**Table 1:** Strain gauge sensor positions on the stress crossbar in the local XYZ coordinate system (see Figure 1c). Sensors are annotated as MP (measuring point).

Local Coordinate of the Crossbar						
X (% of H)	Y (Face)	Z (% of B)				
		−27	−18	−9	0	18
13	1				MP01	
	4		MP11		MP07	
69	4				MP08	
81	1		MP04		MP02	
	2				MP05	
	3				MP06	
	4	MP13	MP12	MP10	MP09	MP14
91	1				MP03	

**Table 2:** Temperature sensor positions on the temperature crossbar in the local XYZ coordinate system (see Figure 1c). Sensors are annotated as MP (measuring point).

Local Coordinate of the Crossbar							
X (% of H)	Y (Lamella)	Z (% of B)					
		−45	−36	−27	−18	0	18
2	2nd	MP28	MP26		MP23	MP18	MP30
13	1st					MP15	
	2nd					MP19	
69	2nd					MP20	
81	1st				MP17	MP16	
	2nd	MP29	MP27	MP25	MP24	MP21	MP31
91	2nd					MP22	

## Data Availability

Not applicable.
